# Operative Exposure of a Surgical Trainee at a Tertiary Hospital in Kenya

**DOI:** 10.1155/2015/724506

**Published:** 2015-09-13

**Authors:** Daniel Kinyuru Ojuka, Jana Macleod, Catherine Kwamboka Nyabuto

**Affiliations:** ^1^Department of Surgery, University of Nairobi, P.O. Box 19969-00202, Nairobi, Kenya; ^2^Department of Surgery, Kenyatta University, Kenya; ^3^Spinal Injury Hospital, Nairobi, Kenya

## Abstract

*Background*. Psychomotor domain training requires repetitive exposure in order to develop proficiency in skills. This depends on many training factors in any training institution. *Objective*. This study sought to look at the operative exposure of surgical trainees in a tertiary hospital in a developing country. *Design and Setting*. This was a six-month retrospective study performed in one surgical firm at Kenyatta National Hospital. *Patients and Methods*. The files of all patients admitted to the unit at that time were retrieved. The demographics, diagnosis at admission, need for surgery, and cadre of operating surgeon among others were recorded. Scientific Package for Social Sciences (SPSS) version 17.0 was used for data entry and analysis. *Results*. The study cohort was 402 patients of the 757 patients admitted in the study period. The average age was 36.7 years, a female to male ratio of 1 : 2.5. The majority (69.7%) of patients required surgery. Trauma was the most common reason for admission (44.5%). Year 2 residents received the most clinical exposure. Consultant was available in only 34.5% of the cases. *Conclusion*. The junior residents performed the vast majority of procedures with an unsatisfactory amount of supervision from the senior residents and faculty.

## 1. Introduction

Medical educators have in the last decade delineated the domains of competency expected of graduating general surgery residents [1]. The patient care domain in the American model overlaps with the technical skills domain in the Canadian model, both clearly delineating the need for competency to perform certain operative procedures as part of the graduate surgical trainees' skills at the end of their training. Repeated purposeful practice in performing complex psychomotor tasks like surgical operations has been shown to be of paramount importance in achieving this level of competence [2].

In the last 5 years, the surgical education literature has documented a reduced level of operative exposure for skill acquisition for graduating general surgery trainees in England and North America [3–5]. However, the operative exposure and the operative skill development opportunities for Kenyan surgical trainees have not been investigated. The surgical trainee examining board in the United States of America, the American Board of Surgery (ABS), as well as program directors and residents in the American training systems have recorded serious concerns regarding the gap between surgical knowledge and surgical operative experience with a resultant reduced readiness for practice in general surgery [5] Approximately 80% of graduates of general surgical residency programs in an American training system now opt for additional postresidency fellowship training, often citing lack of confidence to start practise immediately as the main reason for the fellowship [6]. This change in surgical trainee operative confidence level at the end of training is often cited to be a result of the reduction in operative exposure for these surgical trainees as well as an increased focus on patient safety and the consultant surgeon's need for accountability. The situation of the graduating surgical trainee in the Kenyan context has not yet been similarly objectively elucidated.

Therefore, we designed a study to analyse the operative exposure and hence skill development opportunities of Kenyan surgical residents in a six month period in a surgical ward within a referral and teaching hospital in Nairobi, Kenya.

## 2. Patients and Methods

This was a six-month retrospective study between March 2012 and August 2012 in a single general surgical firm of the main referral and teaching hospital in Kenya that trains University of Nairobi surgical trainees (KNH). The trainees have rotations of 3 months each and so during the study time, there were 2 rotations with two different groups of residents. The call system of the hospital is that the resident takes a weekly call of seven days straight in the general surgical wards. This call week will have a maximum of three days of admission. There is no statutory regulation regarding duty hours for doctors in the country because of the inadequate number of doctors. The number of consultants in this unit during the study was 8. The ward has a bed capacity of 45. The residents working in the ward are distributed at random from year 2 to year 5 with the exception of year 3 residents who usually work in the speciality units. The total number of residents working at any one given time depends on the total number of residents available. The surgical patients come to the wards and for surgery in a variety of ways. Some emergencies will have been referred from other institutions as well as those who come to KNH directly for emergency care. Elective patients are chosen by the residents in consultation with the faculty for the purposes of learning. They are chosen most commonly from the surgical outpatient clinic run by the unit. But they can also already be in the ward after an emergency admission and have remained there for an extended period of time for a number of different reasons and they now require elective surgery.

Between March and May 2012, we had 6 residents; 5 were postgraduate year (PGY) 2, and one was PGY 4. Between June and August 2012, we had 6 residents; 3 were PGY 2, one was year 4, and two were year 5. The learning of the resident is expected to take place as they are exposed to patients during the admissions and during the operations. Operating exposure can be as the primary surgeon, meaning the main person operating, or can be as assistant surgeon. During this time, the resident is expected to consult the faculty for input both in decisions regarding the indication for surgery and for nonoperative care of patients. This should be recorded in the patient files. The residents in PGY 3 were operating only on those of head injury and chest injury. This is because acute neurosurgical patients and acute cardiothoracic patients are admitted in general surgical wards but it is the faculty and the residents in those units that will take primary responsibility for those patients. The process of admission and ongoing care of these patients is performed by the residents in the general surgical wards. The calls are taken for a whole week by one resident; it therefore means that, in these 12 weeks, any one resident will have only two call weeks. In any one week, there will be 2-3 days for admitting patients to the unit; given that there are three units, one unit covers the weekend such that weekend calls for each unit are only every three weeks.

Data collection included demographics, diagnosis at admission, admission type, need for surgery, admitting doctor, cancellation of elective surgery, supervision by the faculty, primary surgeon, assistant surgeon, complication, length of stay, and mortality.

These were entered into SPSS version 17 and analysed using frequencies for interval or binary data and means for quantitative data. Differences between frequencies were analysed using the Chi-square statistic or Fisher's exact test as appropriate. Any difference was considered significant if the *p* value was less than 0.05 as per standard statistical convention.

The study was approved by the Kenyatta National Hospital and University of Nairobi Ethical and Research Committee.

## 3. Results

**Figure 1:**
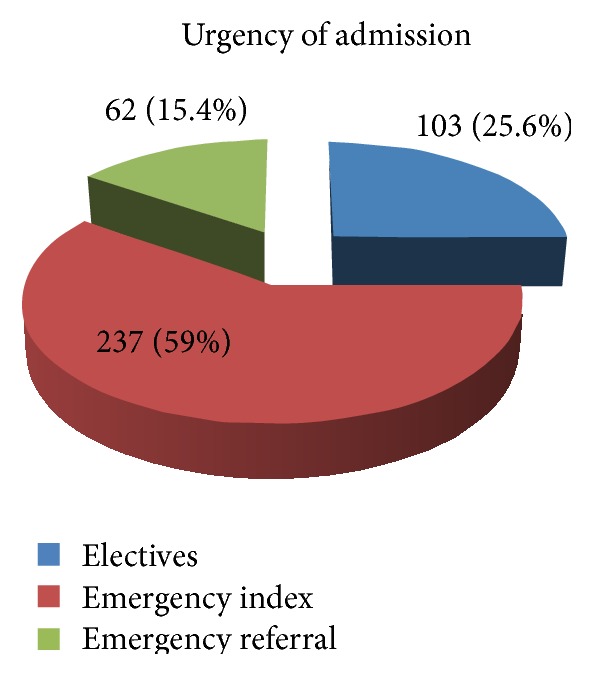
Urgency of admission.

The number of patients admitted to the ward during this time was 757, but the number of files we were able to retrieve was 402, the final study cohort. The mean age of the study cohort (*n* = 402) was 36.7 years; males comprised 71.4%. During this study period, 293 of the 402 patients (79.2%) were scheduled for surgery. Most admissions were emergencies (74.9%) (see Figure 1).

The cancellation rate for scheduled surgeries was 7.8%. The majority of the cancellations were because of project-related surgeries preferentially using operating theaters (meaning instead of routine schedule surgery, external expertise in collaboration with administration and local surgeons organize a period where surgeries for particular subspecialty area for a period). Hence, the regularly scheduled surgery is postponed, often without prior arrangement. The final two main reasons were patients who came to the operating room beyond the accepted finishing time of 3:00 pm (2/8) and nonoptimized patients (1/8).

**Figure 2:**
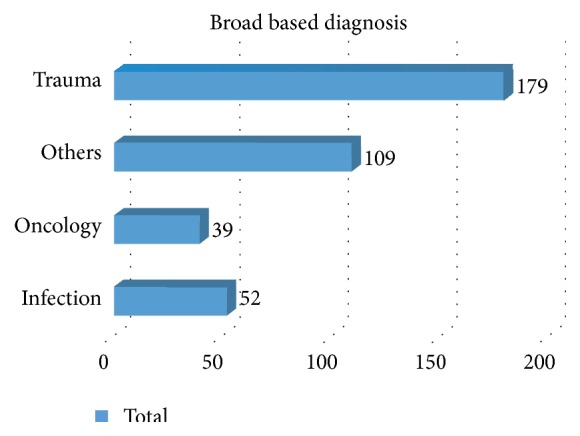
Diagnosis by broad categories.

Trauma was the most dominant reason for admission (179/402, 44.5%) (Figure 2). The main body system injured was the head. The largest four diagnoses and their frequency of occurrence are listed in Figure 1 and then each diagnostic category is further detailed in Tables 1 [Table tab2] [Table tab3]–4.

Urological cancers followed by breast cancer were the frequent cancers, while appendicitis was the most frequent infectious surgical pathology seen and hernia was also common (see Tables 1–4).

Exposure by admission shows that the 8 year 2 students shared 392 admissions, which translate to 49 admissions per student, while the two year 4 students shared 6 admissions translating to 1.5 admissions per student; year 5 students shared 2 admissions translating to 1 admission per student.

**Figure 3:**
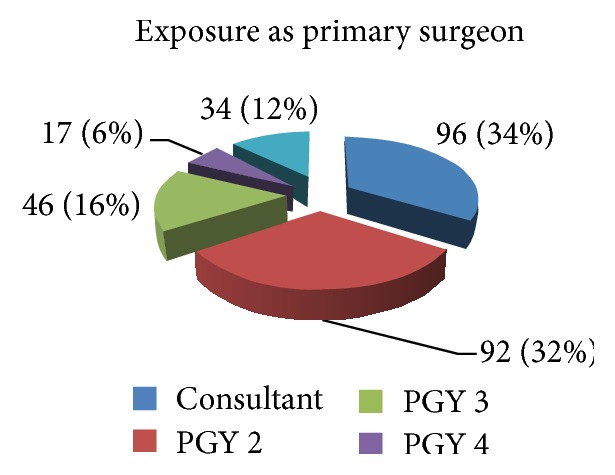
Operating doctor.

Exposure as a primary surgeon in this study shows that the eight PGY 2 residents performed 92 operations, which is equivalent to 11.5 operations per PGY 2 resident, while the two PGY 4 residents performed 17 operations, an average of 8.5 operation per PGY 4 resident, and the most operations per resident were completed by the two PGY 5 senior residents who performed 34 operations which is equivalent to an average of 17 operations per PGY 5 resident in the six-month study period (see Figure 3).

Operative exposure gained by operative assisting demonstrated a decreasing exposure as residents became more senior: per resident operation assistance ratio was 16.5 assists per resident for PGY 2, while it reduced to 11.5 and then to 4.5 for PGY 4 and 5 residents, respectively.

**Figure 4:**
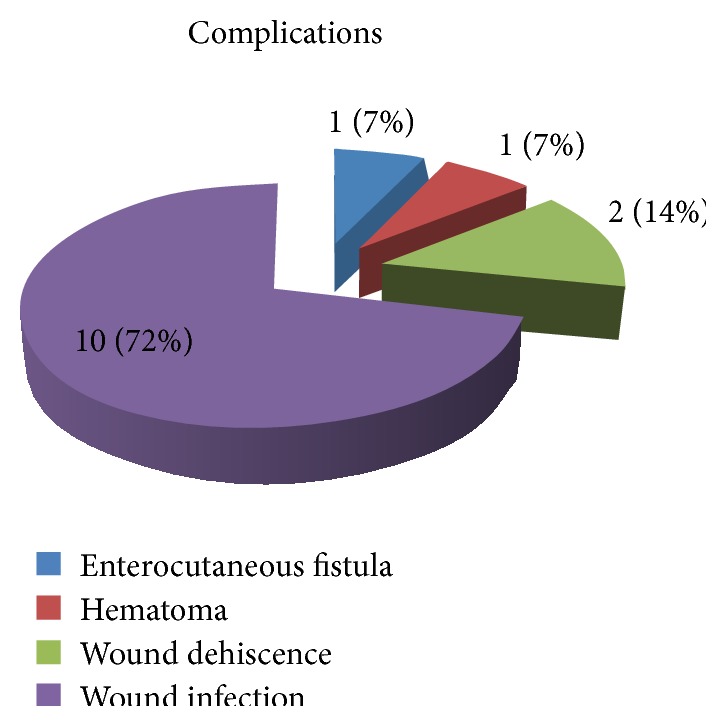
Complications.

Complication rate was 2.8% (14/285) with wound infection being the most common (see Figure 4). The mortality rate for this period was 6.2%.

The consultant was seen, by comments in the file, to supervise and to have been involved in 34.5% of the decisions and assisted in a few cases {8/100 (2.7%)} of all admissions and operations during this period. When the consultants or senior level residents operated, the complications were fewer (*p* value of 0.001). There was no relationship found between the cadre of resident operating and patient's length of hospital stay (*p* value of 0.052). Consultants were the primary surgeons in the majority {81/103 (78.6%)} of elective procedures.

## 4. Discussions

Training as a surgeon is more than just the number of years of training; it involves learning the techniques of operating and the attainment of adequate judgment, overall patient care skills, and professionalism [7].

In Kenya, there has been little system change in the resident training since its inception but the number of trainees in training has increased significantly. In 2009, there was the introduction of a 5-year training curriculum instead of the previous three and half years. Before 2008, the majority of trainees were sponsored by the government for masters of medicine in surgery program (surgery residency training). However, more recently, there has been a dramatic increase in self-sponsored students, who now comprise almost 80% of all the trainees. With these changes, there is a resultant increase in the number of trainees at any particular time in the ward. As a result of this dramatic increase in resident numbers, training experiences such as call have sometimes been reduced to two calls for an entire rotation of 12 weeks. This of course means exposure to fewer patients in terms of both admissions and surgery for residents. This scenario may result in either the resident taking more years in training to meet the required exposure or graduating with less technical experience.

One common approach to learning in residency involves senior residents playing an increasing role in patient care such that the junior students learn and are mentored to a large extent by the seniors as well as consultants. However, in this study it appears that this form of learning for the juniors was unavailable given that we found most senior residents were not present in all or most of the surgeries. In this study, we found that the students in year 2 were actually assisted most often by fellow year 2 students. A solution to this situation may be to introduce a chief residency system.

There were a larger number of PGY 2 residents compared to other years limiting our analysis such that we were unable to compare the exposure between the different years of residency training. This discrepancy in numbers was because of a new curriculum that introduced a large number of trainees in one year, resulting in this larger number of PGY 2 residents. Therefore, our results of increased exposure to admissions and surgeries by postgraduate year 2 residents may in fact be simply a reflection of the absolute numbers of the postgraduate year 2 residents as opposed to a reflection of a type of training system.

Exposure by type of admission demonstrates few elective patients (25.6%). The disadvantage of this in a learning institution is that it means the students have less exposure with the consultant because most of the emergencies are operated without assistance from consultant or at times even without senior residents. In a recent study looking at the changing caseloads for surgical residents, Varley et al. noted that reduced electives exposures should prompt reforms so that the caseloads are taken into account to maintain quality of training [7]. Maddern in an article published about 18 years ago noted that changing times required a requisite change in surgical teaching methods [8]. Our study emphasizes the changes needed are those that will increase the exposure despite the increased number of students with unchanging numbers of patients. One possible solution is to consider introducing the system where chief residents are involved in most of the surgeries both emergency and elective. This way, senior residents take more responsibility in giving service, learning, and teaching. This is the model predominantly followed in surgery resident training systems in North American centers.

We previously reviewed the operative exposure of our residents to emergency surgery but this study is the first to define the elective surgical exposure of our surgery residents [9]. In our previous study, one hundred and forty five patients were admitted. The number of admissions per resident varied between 30 and 41. Operative experience where the resident was the principal surgeon ranged from 11 cases to 23 cases per resident. A second resident assisted in 8 out of the fifty-eight cases operated on and consultant support was infrequent. The previous study was of three-month duration, with a retrieval rate of files at 72%. The duration for the current study is increased but retrieval is less at 53.2%. The admission per student ranges from 1.5 to 49, which demonstrates reduction somewhat, while that of exposure to primary surgery averages at 8.5 to 11.5 per student, and assistance at 4.5 to 16.5 per student. In all areas, this shows reduction, although this probably can be explained more by the lower retrieval rate and increased number of students than by a valid change in exposure per student.

The reduction in numbers in this study is likely reflective of the combination of both elective and emergency surgery together. The PGY 2 resident is meant to perform 15 appendectomies as primary surgeon (The Log Book of department of surgery requirement) in two general surgical rotations that total six months. But in this study of the same duration there were only 20 appendicitis cases in total. The PGY 4-5 residents require 10. In total, one training as a general surgeon requires 25 appendectomies. If the trend above continues, it means that any one PGY 2 resident may not be able to meet the competency requirements to graduate.

This study and our previous study [9] reveal an insignificant level of supervision by the consultant. Though the consultants are involved in elective cases (100%), the same does not hold true during emergency surgery cases. The most critical patient, the emergency surgery patient, is left in the hands of the resident while the least critical patient, elective patients, are operated on by the faculty. The involvement of faculty should improve resident clinical skills and knowledge and, therefore, would be expected to reduce the higher complication rates taking in to account patient acuity.

The limitation of the study is its retrospective nature which results in a reduced retrieval of medical records and the inherent bias of nonrandom record loss as is reflected in a retrieval rate of only 53.2%.

## 5. Conclusion

In conclusion, this study reveals a reduced clinical exposure rate for surgery residents in both elective and emergency surgery volumes. In particular, in the more senior years of training, there is a reduction in surgeries performed with successive years of training. There is a skew toward emergency surgery exposure with a paucity of elective procedures. To add to this finding, the supervision is the highest during elective surgeries (as reflective in consultant involvement) while exposure by residents at all levels is lowest in these same elective procedures. Concurrently, emergency surgeries are the commonest surgeries for all residents but in particular for the junior residents (PGY 2) and are most often assisted by only similar level residents (PGY 2). This study highlights the need for changes that will enable the resident to acquire necessary technical skills for their work as surgeons when they graduate. There is a need for greater involvement by the faculty in the management of surgical emergency patients, especially as they have the greatest morbidity and mortality.

## Figures and Tables

**Table 1:** Trauma.

Diagnosis	Number (*n* = 179)
Head injury	87
Chest	15
Abdominal	28
Soft tissue injury	28
Snake bite	1
Human bite	2
Multiple	18

**Table 2 tab2:** Oncology.

Area	Number (*n* = 39)
Head and neck	4
Gastric	2
Oesophageal	4
Colorectal	3
Urology	21
Gall bladder	1
Cervical	2
Metastasis	2

**Table 3 tab3:** Infections.

Area	Number (*n* = 52)
Appendicitis	22
Cholecystitis	3
Cellulitis	9
Necrotizing fasciitis	5
Peritonitis	6
Pyomyositis	1
Urological	6

**Table 4:** Anatomical defect/inflammation.

Diagnosis	Number (*n* = 109)
Hernia	28
Urethral stricture	12
Pancreatitis	2
Hemorrhoids	6
Goitre	10
Undescended testis	3
Urinary calculi	5
Prostatic enlargement	5
Intestinal obstruction	18
Gall stones	7
Benign breast disease	4
Testicular torsion	1
Enterocutaneous fistula	3
Fistula in ano	2
Anal fissure	3
